# Long‐Term Outcome of Chronic Hepatitis B—Histological Score and Viral Genotype Are Important Predictors of Hepatocellular Carcinoma

**DOI:** 10.1111/jvh.70008

**Published:** 2025-01-29

**Authors:** Anders Eilard, Johan Ringlander, Maria E. Andersson, Staffan Nilsson, Gunnar Norkrans, Magnus Lindh

**Affiliations:** ^1^ Department of Infectious Diseases, Institute of Biomedicine, Sahlgrenska Academy University of Gothenburg Gothenburg Sweden; ^2^ Department of Infectious Diseases Sahlgrenska University Hospital Gothenburg Sweden; ^3^ Department of Laboratory Medicine, Institute of Biomedicine University of Gothenburg Sweden

**Keywords:** hepatitis B virus, hepatocellular carcinoma, histology, prognosis, risk factors

## Abstract

Current guidelines to prevent hepatocellular carcinoma (HCC) by chronic hepatitis B virus (HBV) infection are based on risk assessments that include age, sex, and virological and biochemical parameters. The study aim was to investigate the impact of predictive markers on long‐term outcomes. The clinical outcomes of 100 patients with chronic hepatitis B were investigated 30 years after a baseline assessment that included liver biopsy. A favourable outcome—HBsAg loss or HBeAg‐negative infection (ENI; previously termed ‘inactive carrier’)—was observed in 74% of all patients, whereas 7% developed HCC. HBsAg loss was observed in 75% of patients with genotype A, compared with 42%, 33% and 0% with genotypes D, B and C, respectively (*p* < 0.0001). HCC developed in 3 patients (33%) with genotype C as compared with 3 (17%), 1 (2%) and 0 patients with genotypes B, D and A, respectively (*p* < 0.0001). In multiple logistic regression analysis, both HBsAg loss and HCC were associated with HBV genotype and baseline HBV DNA level, and HCC also with histological score. The results suggest that genotyping and histological assessment may improve outcome prediction and help decisions about HCC screening, particularly in populations with HBV‐infected individuals of mixed geographic origin.

## 1 Introduction

Chronic hepatitis B virus (HBV) infection is a major cause of liver cirrhosis and hepatocellular carcinoma (HCC) [1, 2]. The course of the infection is highly variable, and the main task for clinicians is to identify patients who are at risk for complications, in order to prevent necroinflammation and HCC by antiviral therapy and identify curable HCC by screening programs. Current guidelines recommend treatment for patients with significant inflammation and viral replication, considering other risk factors such as sex, age, geographical origin, and family history of HCC [3, 4], and the indication for treatment has become broader over time. This shift has been motivated by East Asian studies reporting a strong link between HBV DNA levels and both cirrhosis [5] and cancer [6], as well as studies showing cancer risk reduction by nucleoside analogue (NUC) treatment [7, 8, 9, 10, 11].

Previous studies have shown that the risk of cirrhosis or HCC is associated with prolonged elevation of liver transaminases (ALT) [12], male sex and older age and scoring algorithms have been developed for prediction of HCC [13, 14, 15]. Alternative scores, which do not include HBV DNA levels, have been developed for both Caucasian [16] and East Asian [17] patients on NUC treatment. Current guidelines recommend HCC surveillance by liver sonography for patients based on a combination of risk factors such as age, sex, ethnicity and virological tests but the identification of patients for screening is still often difficult. Moreover, the sensitivity of liver sonography is suboptimal for diagnosis of early HCC and magnetic resonance imaging (MRI) has been proposed as a better surveillance tool [18].

It is not known if histological assessment of inflammation and fibrosis can improve identification of patients with a high HCC risk in the absence of liver cirrhosis since most long‐term, large‐scale studies did not consider histopathology, only presence of liver cirrhosis based on clinical data or sonography.

The utility of HBV genotype for risk evaluation is also incompletely known. Genotype C is associated with extended duration of HBeAg positivity, more severe inflammation [19] and a greater risk of liver cirrhosis and HCC [20, 21, 22]. Genotype A has been associated with better outcome [23] and in a previous 10‐year follow‐up study, we found a higher rate of HBsAg loss among patients with genotype A [24].

Seroclearance, defined as the loss of HBsAg from serum, implies a markedly reduced risk for future complications and is an important goal for the treatment of HBV [25]. Published data have shown HBsAg loss rates of approximately 1% per year; however, there are significant differences between studies and populations and lower rates in HBeAg‐positive subjects [26], but differences between genotypes are not well known.

The present study aimed to investigate the impact of HBV genotype, histopathology, and HBV DNA level at baseline on outcomes such as HBsAg loss or HCC.

## 2 Methods

### 2.1 Setting and Participants

A cross‐sectional study conducted in 1993–1995 investigated 160 patients with chronic HBV infection at the Infectious Diseases Clinic of Sahlgrenska University Hospital, Gothenburg, Sweden. Participation in that study was offered to all patients who were HBsAg positive for more than 6 months and not co‐infected with hepatitis C or D viruses, or HIV [27]. Most of them have since then had follow‐up at this clinic, and some have initiated antiviral treatment or HCC screening according to the guidelines. The present, retrospective study included patients with virological and biochemical data from at least one time point between 2000 and 2023. The baseline characteristics of these 100 patients are presented in Table 1, along with the data for the 60 patients from the original study that were not included due to lack of data.

**TABLE 1:** Baseline characteristics of 100 patients with chronic HBV infection included in the present long‐term follow‐up study compared with 60 without follow‐up data (usually because they had moved elsewhere or were deceased).

	Included (*n* = 100)	Not included (*n* = 60)
Sex	61/39	44/16
Age, mean (range)	35.9 (18.8–60.6)	33.8 (17.4–66.6)
Geographic origin		
North Europe	24	6
South Europe	14	7
Middle East	34	24
East Asia	25	11
Africa	3	12
HBeAg^+^ (%)	23 (23%)	13 (22%)
HBV genotype (% HBeAg^+^)		
A	20 (10%)	12 (17%)
B	18 (28%)	4 (75%)
C	9 (67%)	5 (60%)
D	53 (19%)	30 (6.7%)
HBV DNA log IU/mL, median (IQR)	3.68 (3.02–5.79)	3.55 (3.08–5.81)
HBsAg log IU/mL, median (IQR)	3.68 (2.95–4.16)	3.55 (3.01–4.46)
Histology activity index, median (IQR)	4 (2–6)	3 (2–6)
ALT/ULN median (IQR)	0.88 (0.58–1.47)	0.72 (0.48–1.18)
Platelet count ×10^9^/L, median (IQR)^a^	211 (184–236)	218 (182–253)
APRI, median (IQR)	0.52 (0.39–0.73)	0.52 (0.33–0.75)
REACH‐B score, median (IQR)	7 (4–8)	7 (4–9)
PAGE‐B score, median (IQR)	8 (4–12)	7 (6–12)

*Note:* APRI (AST to Platelet Ratio Index) = (100 × AST/ULN)/platelet count.

Abbreviations: IQR, interquartile range; ULN, upper limit of normal.

^a^ Platelet count was missing in 2 cases.

### 2.2 Baseline Parameters

The following baseline parameters were recorded: age, sex, HBeAg status, HBsAg level in serum (Architect, Abbott, Chicago, IL, USA), HBV DNA level in serum (Cobas Amplicor or Cobas TaqMan, Roche Diagnostics, Branchburg, CA, USA), HBV genotype, histological activity index (HAI, Knodell score [28]), alanine and aspartate aminotransferase levels (ALT, AST) and platelet counts. HBV DNA levels in copies/mL were translated to IU/mL by division by 5.82 (a conversion factor provided by Roche when the IU was introduced in their assay in 2005). HCC risk scores were calculated based on selected parameters: PAGE‐B [16] (age, sex, platelets) and REACH‐B [13] (age, sex, HBV DNA, HBeAg status and ALT).

### 2.3 Outcome Parameters

Clinical outcomes were divided into four groups: loss of HBsAg, HBeAg‐negative infection (ENI, previously termed ‘inactive carrier’), ongoing long‐term nucleoside analogue treatment (NUC), and hepatocellular carcinoma or liver transplant (HCC). Of note, there were no patients with HBeAg‐positive (EPH) or HBeAg‐negative (ENH) hepatitis who had not initiated NUC treatment. HBV DNA and HBsAg levels at the time of HBsAg loss, initiation of NUC, or diagnosis of HCC or transplantation were also recorded.

### 2.4 Statistics

Statistical analyses of potential associations between baseline parameters (age, sex, HBV DNA level, HBsAg level, HBV genotype, histological activity index and risk scores) and three dichotomous outcomes: (i) HBsAg loss versus no loss; (ii) HBsAg loss or ENI versus NUC or HCC; and (iii) HCC or not HCC. Univariate comparisons were performed using the Mann–Whitney *U* test, Kruskal–Wallis test, and Fisher's exact test, as appropriate. The potential impact of several baseline factors on outcomes was analysed using forward stepwise multiple logistic regression. Kaplan–Meier with left truncation was used to illustrate the impact of genotype on HBsAg loss by age.

### 2.5 Ethics

The study protocol was in accordance with the ethical guidelines of the 2000 Declaration of Helsinki. The original study was approved by the regional ethics board of Gothenburg (Dnr 336/92) and a written informed consent was obtained from each participant. The retrospective investigation of follow‐up data was approved by the Swedish Ethical Review Authority (Etikprövningsmyndigheten, Dnr: 2022‐01628‐01) without requirement of individual consent from participants.

## 3 Results

### 3.1 Baseline Characteristics and Overall Outcomes

At baseline, the mean age was 35.9 years and 23% of the patients were HBeAg‐positive (Table 1). A large proportion of patients had a favourable clinical outcome with either loss of HBsAg (43%) or achievement of ENI (30%), but a significant fraction (7%) developed HCC. These outcomes were dependent on baseline factors such as HBV DNA levels, HBV genotype, and histopathology, as described below. Information about body‐mass (BMI) index and alcohol consumption was incomplete, but available data suggest that less than 10% were obese and less than 5% heavy drinkers. Diabetes at baseline or during the follow‐up period did not differ significantly between the groups (13%, 13%, 6% and 14% in the HBsAg loss, ENI, NUC and HCC groups, respectively).

### 3.2 HBV DNA Levels

**FIGURE 1:**
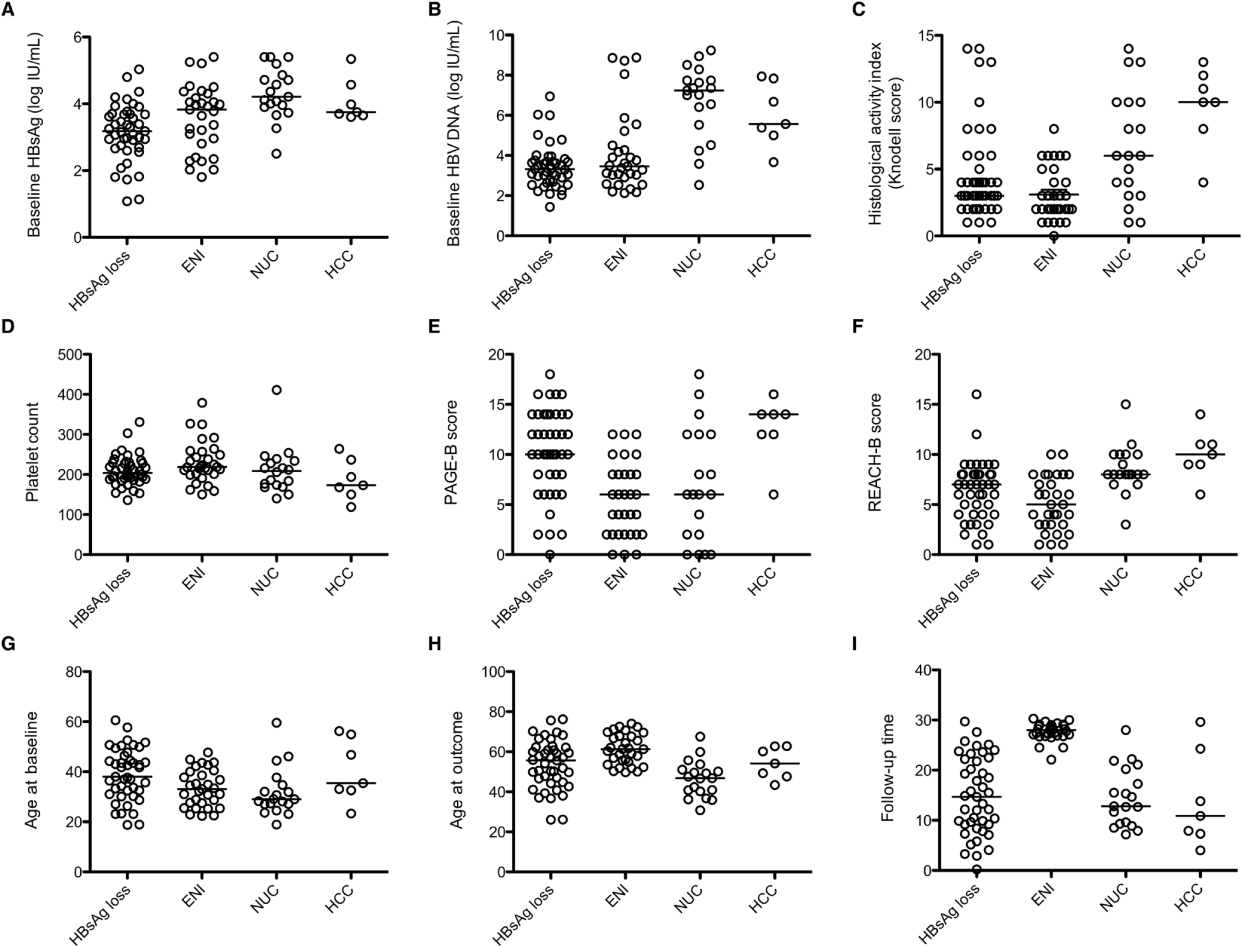
Individual (A–D, G) and combined (E, F) baseline factor values in 100 patients with different long‐term outcomes (HBsAg loss, HBeAg negative infection (ENI, as defined in EASL guidelines), NUC treatment or HCC). (H, I) shows age at outcome and interval between baseline and outcome.

A lower serum level of HBV DNA at baseline was strongly associated with HBsAg loss, and a higher level was associated with HCC, as shown in Tables 2 and 3 and Figure 1. Out of 60 patients with HBV DNA below 4.0 log_10_ IU/mL at baseline, 36 (60%) achieved HBsAg loss and 21 achieved ENI (35%), whereas two were on long‐term NUC and one developed HCC. The two patients with low baseline HBV DNA levels who were NUC‐treated had low baseline histology scores (HAI 3), were infected with genotype B, and started NUC because of reactivation with elevated ALT and HBV DNA levels of 5.48 and 6.74 log_10_ IU/mL, respectively, 17 and 15 years after the baseline evaluation. One patient who developed HCC had HBV DNA of only 3.67 log_10_ IU/mL at baseline, but also had elevated ALT levels and severe inflammation and fibrosis on biopsy, and on several subsequent time points the HBV DNA levels were high (5–8 log_10_ IU/mL).

**TABLE 2:** Baseline factors and clinical outcomes.

	HBsAg loss	ENI	NUC	HCC
	43	31	19	7
Sex (M/F)	33/10	13/18	10/9	6/1
Age (mean years, SE)	38.8 (1.6)	33.4 (1.3)	31.7 (2.2)	40.3 (4.7)
HBeAg^+^ (*n* = 23)	2	4	14	3
HBeAg^−^ (*n* = 77)	41	27	5	4
HBV genotype				
A (*n* = 20)	15	4	1	0
B (*n* = 18)	6	5	4	3
C (*n* = 9)	0	2	4	3
D (*n* = 53)	22	20	10	1
HBV DNA (median IU/mL)	3.32	3.46	7.24	5.56
HBsAg (median IU/mL)	3.18	3.83	4.21	3.75
Platelet count (median ×10^9^/L)^a^	204	219	209	173
ALT/ULN, mean (SE)	1.26 (0.28)	1.16 (0.26)	1.61 (0.21)	2.51 (1.12)
APRI, mean (SE)	0.67 (0.14)	0.57 (0.70)	0.85 (0.15)	1.18 (0.33)
Histology activity index, median (IQR)	3 (2–6)	2 (2–5)	6 (3–10)	10 (8–12)
Inflammation score	2 (2–3)	2 (1–3)	5 (2–7)	7 (5–9)
Fibrosis score	1 (1–3)	1 (0–1)	1 (1–3)	3 (3–4)

*Note:* APRI (AST to Platelet Ratio Index) = (100 × AST/ULN)/platelet count. Histology activity index, HAI, is the sum of liver inflammation and fibrosis scores, which both are ordered category scores.

Abbreviations: SE, standard error; ULN, upper limit of normal.

^a^ Platelet count was missing in 2 cases.

**TABLE 3:** Impact of baseline factors on three outcomes.

	HBsAg loss				NUC or HCC				HCC			
	OR	*p*	OR_adj_ ^a^	*p*_adj_ ^a^	OR	*p*	OR_adj_ ^a^	*p*_adj_ ^a^	OR	*p*	OR_adj_ ^a^	*p*_adj_ ^a^
Male sex	3.4	0.004	8.0	0.003	1.03	0.95			4.15	0.14		
Age at baseline	1.06	0.01			0.97	0.26			1.05	0.23		
HBeAg^+^	0.12	< 0.001			21.4	< 0.001	22.2	< 0.001	2.74	0.23		
HBV genotype		< 0.001		0.04		< 0.001		< 0.001		0.004		< 0.001
A (*n* = 20)	4.2		5.6		0.2		0.01		0		0	
B (*n* = 18)	0.7		0.44		2.4		2.12		10.4		36	
C (*n* = 9)	0		0		13.4		4.61		26		23	
D (*n* = 53)	ref		ref		ref		ref		ref		ref	
HBV DNA (log_10_ IU/mL)	0.56	< 0.001	0.52	0.004	2.15	< 0.001			1.43	0.046		
HBsAg (log_10_ IU/mL)	0.41	< 0.001	0.47	0.048	3.56	< 0.001			1.89	0.14		
Platelet count^b^	0.99	0.32	0.98	0.012	0.99	0.21			0.98	0.07		
Histology activity index^c^	0.96	0.51			1.30	0.001	1.54	< 0.001	1.38	< 0.001	1.92	< 0.001

^a^ Adjusted using multiple logistic regression analysis.

^b^ Platelet count was missing in 2 cases.

^c^ An ordered category score that combines inflammation and fibrosis.

The median HBV DNA levels had declined by 2.93 log_10_ IU/mL after median 15 years in the patients that achieved HBsAg loss, and by 2.15 IU/mL after median 28 years in those that had ENI as outcome (*p* < 0.0001 for both), whereas no significant decline occurred in patients who had started long‐term NUC (after median 15 years) or developed HCC (after median 12 years).

### 3.3 HBeAg Status

In total, 23 patients were HBeAg‐positive at baseline. As shown in Table 1, HBeAg positivity differed between genotypes: 10% in those infected with genotype A and 67% in genotype C. As shown in Table 2, 26% (6/23) of HBeAg‐positive patients compared with 88% (68/77) of HBeAg‐negative patients achieved HBsAg loss or ENI, a difference that was linked to other baseline factors, such as age, genotype, and HBV DNA level.

### 3.4 HBsAg Levels

**FIGURE 2:**
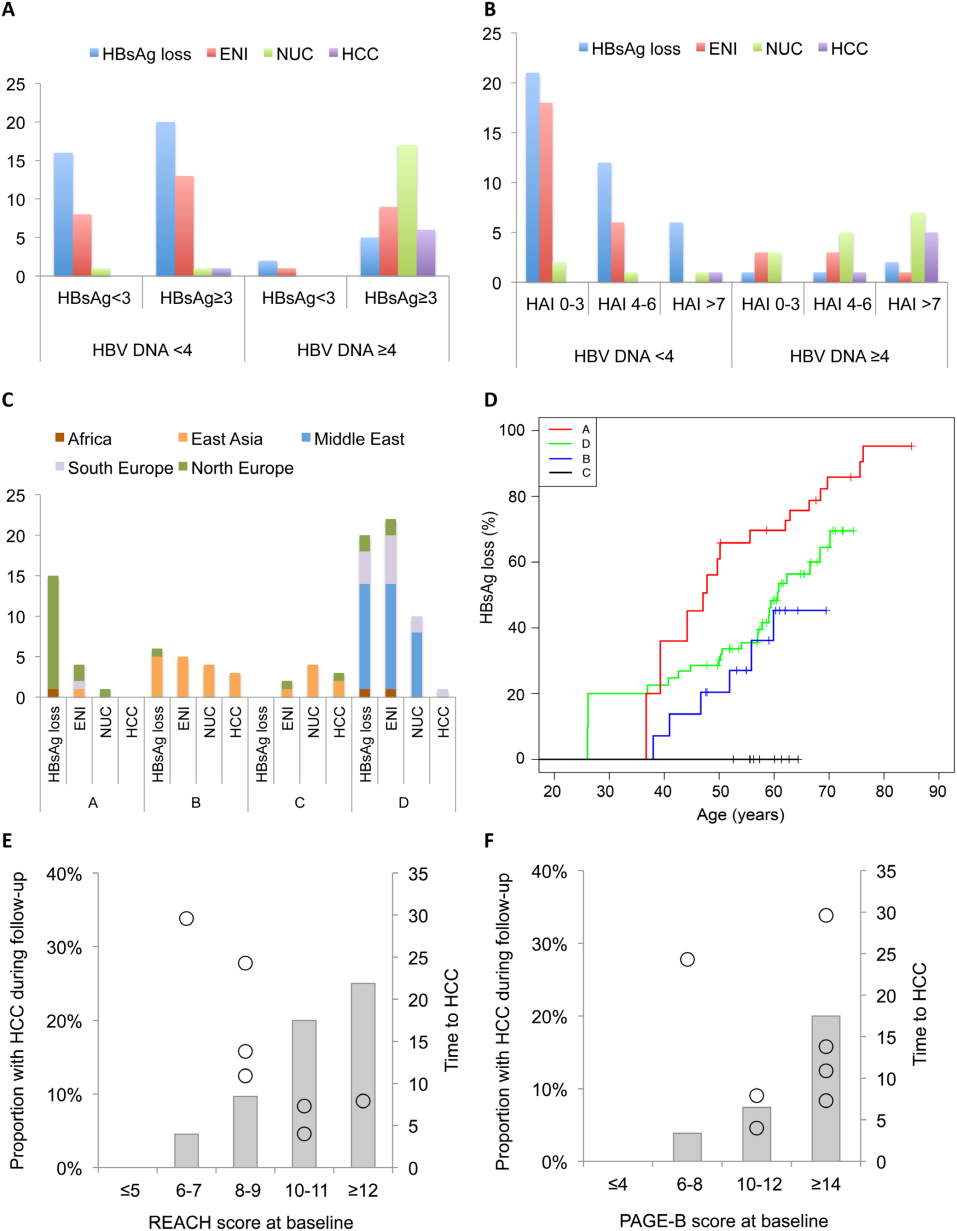
(A, B) shows clinical outcome by combinations of (A) HBV DNA level and HBsAg level (log_10_ IU/mL) at baseline, or (B) HBV DNA level (log_10_ IU/mL) and HAI score at baseline. (C) shows the association between geographic origin, HBV genotype and clinical outcome. (D) Kaplan–Meier plot with left truncation illustrating the impact of genotype on the achievement of HBsAg loss by age. Lower histograms show the proportion of patients with different (E) REACH‐B or (F) PAGE‐B scores that developed HCC. Open circles show the time from baseline to HCC for each of the 7 patients, illustrating that REACH‐B (but not PAGE‐B) scores were inversely associated with the time to diagnosis of HCC.

Lower serum HBsAg levels at baseline were associated with HBsAg loss or ENI achievement, as shown in Table 3. As shown in Figure 1B, out of 28 patients with HBsAg below 3.0 log_10_ IU/mL at baseline, 18 (64%) achieved HBsAg loss, 9 (32%) had ENI as an outcome, one was on long‐term NUC, and none developed HCC. Combining HBsAg and HBV DNA levels only marginally improved the prediction of outcomes, as shown in Figure 2A. At outcome, the HBsAg levels had declined by 4.15, 1.67, 0.65 and 0.93 log_10_ IU/mL in patients with HBsAg loss, ENI, NUC and HCC, respectively, after a median of 15, 28, 13 and 11 years.

### 3.5 HBV Genotype

Tables 2 and 3 show that among the 20 patients with genotype A, 75% showed HBsAg loss and 20% had ENI at follow‐up. In 53 patients with genotype D, loss of HBsAg was achieved in 42% and ENI in 38%, 19% were on long‐term NUC treatment, and one patient developed HCC (5.6%). Among 18 patients with genotype B, 33% lost HBsAg, 28% reached ENI, 22% were on NUC, and 17% developed HCC. None of 9 patients with genotype C achieved HBsAg loss, 22% reached ENI, 44% were on long‐term NUC, and 33% developed HCC. The Kaplan–Meier plot of the impact of genotype on the achievement of HBsAg loss by age showed relatively similar patterns for genotypes A, B and D, although the rate was slightly steeper for genotype A (Figure 2D). As shown in Figure 2C, the HBV genotype was strongly associated with geographic origin.

Three of the patients with genotype A who achieved HBsAg loss had received a 6‐month interferon‐alpha treatment 2, 5 and 7 years earlier.

### 3.6 Histology Score

The histology activity index (HAI) is an ordered category score that combines inflammation and fibrosis [28]. The HAI (*p* < 0.001) as well as the individual inflammation (*p* = 0.004) and fibrosis (*p* = 0.002) scores were associated with the NUC or HCC outcomes in univariate analysis. HAI was associated with HCC in multiple logistic regression analysis that also included the genotype or the HBV DNA level as independent parameter (the low number of HCC cases limited the number of putative explanatory parameters in the model). Of the seven patients who developed HCC, six had high HAI scores at baseline, including four with severe fibrosis (F3 stage) and pronounced inflammation and two with cirrhosis. One patient, aged 23 years, had an HBeAg‐positive infection without significant liver damage at baseline and developed HCC 24 years later. Figure 2B shows that a combination of HAI and HBV DNA improved the prediction of outcome compared with these two baseline factors alone.

### 3.7 Sex

HBsAg loss was observed in 53% (33/61) of males as compared with 26% (10/39) of females (OR = 3.41, *p* = 0.007). HCC developed in 9.8% (6/61) of males as compared with 2.6% (1/39) of females (OR = 4.15, *p* = 0.24).

### 3.8 Age

The median age at baseline was 38.0 years in patients who achieved and 32.0 years in those who did not achieve HBsAg loss. The median baseline age of patients who did or did not develop HCC was similar (35.4 vs. 34.3 years), and baseline age was not associated with HCC in multiple regression analysis (*p* = 0.23). The HCC cases were identified at ages 43–64 years (median, 54 years), 4–30 years (median, 11 years) after the baseline assessment (Figure 1H,I).

### 3.9 Multivariable Analyses

Multiple logistic regression analysis with a forward stepwise setting was performed with a broad set of baseline factors as independent variables and three dual outcomes as dependent variables: (i) HBsAg loss versus no loss; (ii) HBsAg loss or ENI versus NUC or HCC; and (iii) HCC or no HCC. In these analyses, HBsAg loss was significantly associated with male sex, HBV genotype, lower HBV DNA levels and lower HBsAg levels at baseline, as shown in Table 3. HCC was significantly associated with histological score, HBV genotype and HBV DNA level.

### 3.10 Risk Scores and Outcome

In the univariate analysis, the REACH‐B score (based on age, sex, ALT, HBeAg status and HBV DNA) was associated with NUC or HCC outcome, whereas the PAGE‐B score (based on age, sex and platelets) was only associated with HCC (Figure 1H,I). The REACH‐B score, but not the PAGE‐B score, was associated with the time from baseline to HCC diagnosis (Figure 2E,F).

**FIGURE 3:**
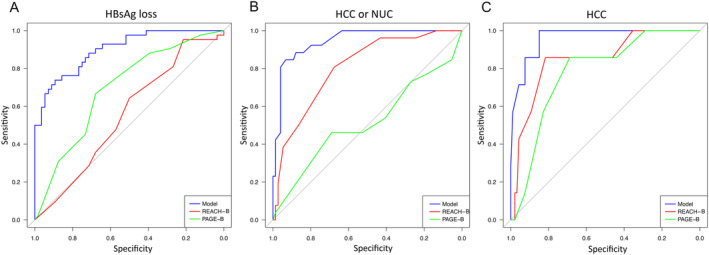
ROC curves comparing prediction of three outcomes (A–C) by risk scores (REACH‐B or PAGE‐B) or by a model that includes the baseline factors that were significant predictors in multiple logistic regression in A (sex, HBV genotype, HBV DNA level, HBsAg level, platelets) and B (HBV genotype, HBV DNA level, histopathology score), and only the two strongest predictors in C (HBV genotype and histological score).

An HCC risk score should have both high sensitivity and specificity, and patients with favourable outcomes, such as HBsAg loss, should not be classified as high risk. To evaluate this, we applied ROC curve analyses for different outcomes. As presented in Figure 3, the ROC curves showed that both PAGE‐B and REACH‐B risk scores were relatively poor predictors of HBsAg loss and relatively good predictors of HCC outcome. However, models based on the baseline parameters that were significantly associated with outcome in the multiple logistic regression analysis were better predictors. Notably, a model based only on HBV genotype and histological score (parameters that are not included in any risk score) was better for HCC prediction than both the REACH‐B and PAGE‐B risk scores.

### 3.11 NUC and HCC

The baseline evaluation was conducted before the introduction of NUC therapy for hepatitis B, and patients with pronounced liver damage (in total 17) were first treated with pegylated interferon‐alpha (IFN). All showed poor or transient responses and were given lamivudine when introduced in the late 1990s, except for three patients with genotype A who had improved after IFN and achieved HBsAg loss 2, 5 and 7 years later. Lamivudine reduced viral load and inflammation, followed by relapse due to resistance mutations within 2–5 years in most patients. When adefovir, tenofovir and entecavir became available, these more effective NUCs were prescribed to treatment‐naïve patients as well as to patients who had been treated with lamivudine or adefovir. Because of these changes in treatment options and indications for treatment, the HCC risk in NUC‐treated patients could be properly evaluated. Only one of the seven patients who developed HCC was on effective NUC and presented with HCC after 18 years of tenofovir treatment. Nineteen additional patients who did not develop HCC had been on effective NUC for an average of 15 years (range 2–23 years). Thus, among the 20 patients (11 males) on effective NUC treatment for a total of 300 years, there was one case of HCC.

## 4 Discussion

This study describes up to 30 years of follow‐up of 100 patients with chronic HBV infection in a low‐endemic country with significant immigration from geographic regions with a higher prevalence of HBV. Most of the patients had favourable clinical outcomes with either loss of HBsAg (43%) or achievement of ENI (30%), but a 7% developed HCC.

The difference in outcome was strikingly associated with the HBV genotype. Patients with genotype A had the best outcome (75% HBsAg loss, 20% ENI), and the outcome for patients with genotype D was also good (42% HBsAg loss, 38% ENI), although a significant proportion (19%) had started NUC therapy and one (1.9%) had developed HCC. In contrast, the outcomes for genotypes B and C were poor, as 22% developed HCC and 30% had long‐term NUC. The poor outcome for genotype C patients, of whom 67% were HBeAg‐positive at baseline—33% developed HCC, and none achieved HBsAg loss—is in agreement with reports of more pronounced necroinflammation [19] and higher risk of developing cirrhosis or HCC [19] as compared with genotype B. To our knowledge, better outcomes for genotypes D and A than for genotypes B and C have not been reported before. The good outcome for genotype A fits with earlier studies on European ‘HBsAg carriers’ of whom a large proportion probably were genotype A infected [29, 30, 31], and the difference versus genotype D is in line with data from Spain [23]. The favourable outcome for a majority of our patients with genotype D agrees with studies in Greece and Italy of individuals with low active infection [32, 33].

Since patients with different genotypes also differ in geographic origin [34], some of the outcome discrepancies might be due to differences in age and mode of infection or host genetics rather than the HBV genotype itself. Patients with genotypes B and C typically become infected at birth, whereas this is probably rare among Western European patients with genotype A, some of whom in our cohort had become infected later in life through sexual transmission. In many African countries, the risk of HCC is high, and studies from South Africa indicate that subtype A1 may have a higher risk of HCC than subtype A2, which probably was predominant among our patients. However, cofactors such as aflatoxin [35] and HIV co‐infection are also believed to contribute to the high rates of HCC observed in African countries [36]. Among our patients with genotype D, both the age at acquisition and the mode of transmission were unknown, but likely including both vertical and horizontal transmission.

Our findings agree with a previous report on the strong impact of HBV DNA levels on the risk for HCC [6]. Thus, we found a strong association between higher HBV DNA levels and the risk of HCC, but also an association between lower HBV DNA levels at baseline and the likelihood of HBsAg loss. Among the 60 patients with baseline HBV DNA levels below 4.0 log_10_ IU/mL, 60% experienced HBsAg loss. One of the 60 patients with low baseline HBV DNA levels developed HCC, but that patient exhibited HBV DNA levels above 5 log_10_ IU/mL shortly after baseline evaluation. As in a previous study [37], adding HBsAg quantification did not improve prediction of the HCC, which however has been reported for patients with low HBV DNA levels in another study [38].

The histological activity index (HAI), a score based on both inflammation and fibrosis, was strongly associated with outcome, and combining HAI scores and HBV DNA levels improved predictions. Thus, an HBV DNA below 4.0 log_10_ IU/mL in combination with an HAI ≤ 3 was strongly associated with a favourable outcome. Furthermore, a ROC curve based only on genotype and histological score predicted HCC better than the REACH‐B score, which is based on age, sex, HBeAg status, HBV DNA and ALT. Previous studies have shown associations between severe inflammation and outcomes in terms of cirrhosis or HCC [39], but the use of liver biopsies in clinical assessments has despite this declined, probably because of the increasing use of elastography [40], and because histopathology is usually not required for the decision to initiate NUC therapy. However, the other important clinical decision, whether to initiate HCC screening (currently comprising sonography every 6 months), is often difficult even with access to practical guidelines [3, 4] and HCC risk scores. Our findings suggest that better predictions of HCC might be achieved if liver biopsies were included in the clinical assessment, as also supported by a long‐term follow‐up study in South Korea [41]. Further studies are required to investigate if liver biopsies improve HCC risk assessment and, if so, if that can be used for individualised and better HCC screening strategies, for example the use of magnetic resonance imaging (MRI) for patients with particularly high risk. MRI screening has been proposed because screening with sonography twice a year might not identify HCC early enough for curative treatment [42], as indicated by recent meta analyses [43, 44].

In agreement with previous reports [12, 13, 45], HCC was associated with the male sex. Interestingly, males also had a higher rate of HBsAg loss (77% vs. 51%), a finding reported in one study [46], but not in others [47, 48].

Several risk scores have been developed to predict HCC in treatment‐naïve patients. In the present study, the REACH‐B score (combining sex, age, ALT, HBeAg status and HBV DNA) was associated with HCC and inversely correlated with the time to diagnosis of HCC. The PAGE‐B score, which was developed to assess HCC risk in patients undergoing NUC treatment, also predicted HCC relatively well in our patients who were not on NUC at baseline, but with a lower accuracy than REACH‐B (high scores were seen also for many patients with favourable outcomes) and without any association with the time to HCC diagnosis.

The main strengths of our study, which make it unique, are the long follow‐up period and the mixed composition of patients, allowing for comparison between the four main HBV genotypes (A–D). The study also has limitations. The duration of HBV infection was unknown for most patients, a limitation that our study shares with most other studies. The number of patients with genotypes B and C was low; therefore, the findings for these genotypes may not be representative. As mentioned, the overlap between genotype, geographic origin and epidemiology indicates that genotype differences might, to some extent, reflect other factors, such as age at acquisition of the infection or genetic differences in host immune responses. The possibility that the genotype itself is important is supported by previously identified differences between genotypes B and C that occur in the same populations. However, further investigation of genotype differences is important, and studies in regions where genotype A is present in populations, together with genotypes B or C, such as the Philippines [49], would be of particular interest. The application of the histological parameter as a numeric score based on the combination of fibrosis and inflammation in our analyses might be questioned. However, this limitation also applies to separate scores for inflammation and fibrosis, which are also ordered categories, and we believe that it is rational to use a combined score for prediction of HCC, which can be assumed to depend on both past (fibrosis) and present (inflammation) damage to liver cells. Finally, it should be mentioned that the REACH‐B score does not claim HCC prediction for longer than 10 years [15] and that the PAGE‐B score was developed for HCC prediction in HBeAg‐negative patients on NUC treatment and not for the long‐term risk addressed in this study [16].

In summary, this long‐term follow‐up study of patients with chronic hepatitis B revealed a strong impact of both histopathology and HBV genotype on clinical outcomes, suggesting that genotyping and liver biopsy may improve decisions about follow‐up and HCC surveillance. Additional long‐term follow‐up studies are required to corroborate our findings, and to evaluate if the addition of genotype and histopathology to the risk assessment might improve the identification of patients who do not need HCC screening at all and of high‐risk patients who might benefit from screening with magnetic resonance imaging.

## Conflicts of Interest

The authors declare no conflicts of interest.

## Data Availability

The data that support the findings of this study are available on request from the corresponding author. The data are not publicly available due to privacy or ethical restrictions.
